# Exosomal miR-17-5p promotes angiogenesis in nasopharyngeal carcinoma via targeting BAMBI

**DOI:** 10.7150/jca.30757

**Published:** 2019-10-22

**Authors:** Bingyue Duan, Si Shi, Huijun Yue, Bo You, Ying Shan, Ziyu Zhu, Lili Bao, Yiwen You

**Affiliations:** 1 Department of Otorhinolaryngology Head and Neck Surgery, Affiliated Hospital of Nantong University, Nantong, Jiangsu Province, China; 2 Institute of Otorhinolaryngology Head and Neck Surgery, Affiliated Hospital of Nantong University, Nantong, Jiangsu Province, China

**Keywords:** nasopharyngeal carcinoma, miR-17-5p, exosome, angiogenesis, BAMBI

## Abstract

**Objective:** The purpose of our study is to investigate the role of miR-17-5p in angiogenesis of nasopharyngeal carcinoma and the crosstalk between HUVECs and CNE-2 via exosomes.

**Methods:** Firstly, flow cytometry, cell viability assay, transwell assay, and tube formation were used to explore the role of miR-17-5p in angiogenesis. Then zebrafish model was used to confirm effects of miR-17-5p on angiogenesis. qRT-PCR analysis and Immunofluorescence assay were used to explore the expression of miR-17-5p in NPC tissues and cells compared to the normal control. Besides, *in vitro* assays were used to analyze the biological functions of miR-17-5p in NPC. What's more, *in vitro* and *in vivo* assays were used to detect the function of exosomal miR-17-5p in angiogenesis. Finally, luciferase reporter assay and western bolt were used to determine the relationship between miR-17-5p and BAMBI.

**Results:** We observed that high expression of miR-17-5p promoted angiogenesis in NPC. Also, high expression of miR-17-5p promoted the NPC cells proliferation and migration. To know whether there's any communication between HUVECs and NPC cells, exosomes derived from CNE-2 cells were collected. Further results showed that exosomal miR-17-5p secreted from NPC promoted the angiogenesis. What's more, *in vitro* assays revealed that miR-17-5p targets BAMBI and regulates AKT/VEGF-A signaling.

**Conclusions:** Our study showed that exosomal miR-17-5p derived from NPC cells promotes angiogenesis via targeting BAMBI and regulates AKT/VEGF-A signaling.

## Introduction

Nasopharyngeal carcinoma (NPC), a solid tumor derived from human nasopharynx epithelium, is mainly distributed in Southeast Asia and southern China. It is the most common malignant tumor of the head and neck 1, 2. The major pathogenic factors of NPC include hereditary susceptibility, Epstein-Barr virus (EBV) infection, environmental factors and lifestyle 3, 4.

With the update of treatment methods, the 5-year survival rate of NPC patients has now reached about 50% 5. Nonetheless, local recurrence and distant metastasis are still the main causes of death 6. Then, in order to improve the quality of life and long-term survival rate of NPC patients, we tried to investigate the mechanisms underlying NPC metastasis.

Angiogenesis is the process of forming new vascular sprouts from existing blood vessels, which involves a number of complex steps including endothelial cell activation, proliferation, maturation, migration and the stabilization of new vascular sprouts 7, 8. Besides tissue growth and development, angiogenesis also happens during wound healing and cancer progression 9. Tumor development needs supply of nutrients and oxygen, and removal of metabolic wastes and carbon dioxide. In 1971, Folkman first proposed the idea "tumor growth depends on angiogenesis"^10^. With the production of new blood vessels in nearby normal tissues, the vascular system carries tumor cells and tumor proteins to sustain tumor development and metastasis 11-13. Hanahan showed that angiogenesis, as one of the biological abilities of tumor, could affect the development of tumor, which is an important target and one of the important prognostic indicators of tumor treatment 11. Therefore, exploring the molecular mechanism of angiogenesis will provide targets for NPC metastasis.

MicroRNAs (miRNAs or miRs) are one kind of endogenous, non-coding small single-stranded RNAs composed of about 18∼25 nucleotides 14. They can block messenger RNAs (mRNAs) translation or negatively regulate mRNAs stability and thereby play a central role in regulating gene expression 15. Numerous studies have confirmed that miRNA is involved in almost all cellular processes, and its role in vascular development has also been confirmed 16-18. Meanwhile, microRNAs expression levels have changed in a variety of human pathological conditions, including tumor angiogenesis 19-22.

With the process of cancer research, lots of people have focused on the mediation of miR-17-5p~92 clusters in angiogenesis. As a polycistronic miRNA gene encoding 7 miRNAs, miR-17-5p~92 cluster was initially described as an oncogene cluster, and later demonstrated to drive important physiological responses during development and disease 23, 24. Among this cluster, miR-17-5p is particularly oncogenic and frequently overexpressed in human cancers 25. Since angiogenesis is a multi-step and complex process, it will be of great significance to study the function and mechanisms of miR-17-5p in regulating angiogenesis.

Recent researches have shown that tumor cells can regulate the tumor microenvironment by secreting nanovesicles called exosomes, which are about 30-100 nm in diameter 26. Exosomes, which function mainly by passing on their contents to receptor cells, are membranous vesicles that formed and secreted by living cells. They contain lots of bioactive components such as miRNA, mRNA, proteins, lipids etc., and have a variety of bioactive functions in physiological and pathological conditions as well 27, 28. Particularly related to our research, many studies have focused on investigating the effects of tumor-derived exosomes on angiogenesis^29^, ^30^. It is believed that tumor-derived exosomes can contribute to tumor microenvironment remodeling and release cytokines and other bioactive components acting on surface receptors of endothelial cells, leading to angiogenesis and promoting tumor migration.

In our study, we found that high expression of miR-17-5p can promote the progress of nasopharyngeal carcinoma. Also, exosomal miR-17-5p released from cancer cells may promote tumor angiogenesis by directly targeting BAMBI.

## Materials and Methods

### Cell lines and clinical samples

Three human NPC cell lines (CNE-2, 5-8F, 6-10B), one normal nasopharyngeal epithelial cell line NP69 and Human Umbilical Vein Endothelial Cell (HUVEC) were all cultured in the laboratory of Otorhinolaryngology Head and Neck Surgery, Affiliated Hospital of Nantong University (Jiangsu, China). NPC cell lines were cultured in RPMI-1640 medium containing with 10% fetal bovine serum (FBS) (Gibco, NY, USA). Keratinocyte-SFM medium supplemented with epidermal growth factor (Invitrogen Life technologies, USA) was used to culture NP69 cell line. HUVECs were cultured in EGM-2 Endothelial Cell Growth Medium provided by Shanghai Distributor of Lonza. All cell lines were incubated in a humidity condition with 37 °C and 5% CO2.

The specimens of fresh tissues, which were confirmed by the pathology department, were obtained from the Department of Otorhinolaryngology Head and Neck surgery. The patients included had not received any anti-tumor treatments prior to biopsy. Normal nasopharyngeal tissues were patients suggestive of NPC according to clinical symptoms, but ruled out by biopsy. Participants all agreed with our research and the Ethics Committee of the Affiliated Hospital of Nantong University approved it.

### Transfection and plasmids

We purchased miR-17-5p inhibition plasmid, miR-17-5p plasmid from GENECHEM (Shanghai, China), si-BAMBI and negative control from RiboBio (Guangzhou, China). 6-well, 24-well and 96-well cell culture plates (Corning, NY, USA) were used to culture different kind of cells. According to the suppliers' instructions, we used Lipofectamine 2000 (Invitrogen, Carlsbad, CA) to transfect siRNAs and plasmids into cells respectively. The protein or RNA levels of cells after 60 h of transfection were determined to compare the transfection efficiency.

### Cell proliferation assay and cell cycle analysis

Cell viability rate was determined using Cell counting kit-8 assay. 100 μl per well of cells at a density of 3~5×10^3^ cells/well were cultured onto 96-well plates. Adding a mixture of 10 μl Cell counting kit-8 reagent (BBI Life Sciences) with 90 μl serum-free medium to each well under dark condition, incubating the cells for 1.5 h at 37 °C, and using the microplate reader to measure the absorbance at 450 nm. For cell cycle analysis, using 70% ethanol solution to fix the cells at -20 ℃ for at least 24 h. 1 mg/ml RNase A was then added into the mixture above and incubated the cells for 20min. After that, centrifuged the cells and stained them with 50 μg/ml propidium iodide (PI, Bectone Dickinson, USA) in PBS-Triton® X-100 for 20 min at 4 °C. Using BD FACScan (BD Biosciences, USA) for cell cycle analysis.

### Immunofluorescence assay

Cells were grouped for different purposes. Fixed the cells with 4% paraformaldehyde at room temperature and blocked with primary antibody block buffer. Using primary antibody anti-Ki67 (1: 50, Proteintech) to incubate the cells at 4 ℃ overnight subsequently. After being washed with PBS, Cy3-conjugated secondary antibodies (1: 200, Proteintech) and Hoechst (Sigma-Aldrich Co., USA) were added at room temperature for cell staining. Finally, observed and photographed using the fluorescence microscope.

### Transwell assay

Millipore chambers with 8 μm pore size (Millipore Co., France) were used for cell migration assays. Resuspended the cells using serum-free medium and adjusted the concentration to 1×10^5^ cells. Medium containing 10% serum was added per well into 24-well plates. After put the champers into each well, seeding 200 μl cell suspension onto the upper chambers. Removed the residual cells in the upper chambers and fixed the cells on the undersurface of the membranes with methanol after 18~22 h of incubation. Crystal violet was used to stain the cells at the room temperature. Five random visual fields were calculated using the microscope.

### Western blot

Protein concentration was measured using BCA Protein Assay Kit. 10% SDS-PAGE and PVDF membranes (Millipore Co., France) were used to separate 20 μg of total cellular protein and the protein transfer. We used 5% nonfat milk in TBST buffer to block the membranes. The antibodies used were as follows: anti-BAMBI primary antibody (1 : 1000, Proteintech), anti-E-cadherin primary antibody (1 : 1000, Cell signaling technology), anti-N-cadherin primary antibody (1 : 1000, Cell signaling technology), anti-Vimentin primary antibody (1 : 1000, Cell signaling technology), anti-VEGFA polyclonal antibody (1 : 300, BBI Life Sciences), anti-CD63 (1 : 1000, BBI Life Sciences), anti-p-AKT (1 : 2000, Cell signaling technology), anti-AKT (1 : 2000, Cell signaling technology). We used HRP-tagged secondary antibodies (1: 2000, BBI Life Sciences) for incubation at room temperature for 1.5 hours. ECL reagent (Millipore Co., France) was used to detect the immunoreactivity.

### Enzyme-linked immunosorbent assay (ELISA)

Serum VEGF-A levels were measured in 6 patients with NPC and 6 healthy controls using Human VEGF-A Precoated ELISA Kit (Dakewe Bio-engineering Co., LTD). Human VEGF-A specific monoclonal antibody was precoated onto 96-well plate. The samples and Biotinylated detection antibody were added to the wells subsequently and washed with 1 × Washing buffer. Used dilution buffer (1 ×) to dilute Streptavidin-HRP, added the mixture to the wells for incubation, and visualized HRP enzymatic reaction by TMB according to the instructions.

### RNA extraction and quantitative real-time PCR

Total RNA was extracted from cells and tissue samples using Trizol Reagent (Sigma, USA). The Transcriptor First Strand cDNA Synthesis Kit (Roche, Germany) was used to synthesize cDNA and Taqman Universal PCR Master Mix used to analyse the gene expression. MiR-17-5p and BAMBI primers used for qRT-PCR were gained from Biomics Biotech (Nantong, Jiangsu, China) and were as follows: hsa-BAMBI forward: 5′-ATCGCCACTCCAGCTACATC-3′ reverse: 5′-CTAGAGAAGCAGGCGCTGAG-3′. U6 and GAPDH were used as controls. All reactions were performed at least three times.

### Exosome isolation and purification

We obtained exosomes according to previous descriptions 31-33. The cell supernatant was centrifuged by differential centrifugation at 300 × g, 3000 × g, 6000 × g, 10000 × g, and cell-free supernatant was subsequently ultracentrifuged at 100000 × g for 60 min at 4 °C (XPN-100, Beckman, USA). The products were resuspended and washed twice by PBS without exosomes and confirmed by the ultracentrifugation again. BCA protein assay kit (PIERCE Co., USA) and Total Exosome RNA and Protein Isolation Kit (Invitrogen Life technologies, USA) were used to estimate the content of protein and RNA in exosome pellets. For experiments *in vitro*, 2 μg exosomes were added to about 2 × 10^5^ recipient cells. All the isolated exosomes above were stored at -80 °C.

### Transmission electron microscopy verify

2.5% glutaraldehyde was used to fix the collected exosomes for 2 h, and 100 μl exosome-depleted PBS was used to suspend the exosomes after they were washed and ultracentrifuged. Taking one drop of 20 μl exosomes to a small carbon-coated grid, staining with 3% phosphotungstic acid for 1 min, and finally using transmission electron microscope for exosome observation.

### Exosome tracking

For exosome tracking experiments, we used purified exosomes labeled with PKH67 membrane dye (Sigma, USA). DMEM was used for washing and resuspending the labeled exosomes. 200 μg/ml of these exosomes were then cocultured with HUVECs for 6 h. After that, the HUVECs above were fixed using 4% paraformaldehyde and nuclei were stained with Hoechst. The images were taken using confocal microscope (TCS SP-5, Leica Microsystems, Germany) and analyzed by Leica Application Suite 2.02.

### *In vitro* angiogenesis

We diluted Matrigel (BD Biosciences) 1:1 with cold EGM-2 Endothelial Cell Growth Medium and spread the mixture on 24-well plates. In order to study the formation of capillary-like structures *in vitro*, 8 × 10^4^ HUVECs were seeded on Matrigel per well. After the cells were deformed and adhered to the walls, removed the medium and added different kinds of treated medium for 6 h incubation at 37°C. The results were then obtained and the length of the blood vessels or the number of loops that HUVECs being per well were then calculated.

### Zebrafish and microinjection

The study conformed to the local institutional laws and the Chinese law for the Protection of Animals. Zebrafish embryos of Tg (fli1a: EGFP) line were raised and staged as described^34^. Morpholino antisense oligomer (MO, Gene Tools) was synthesized according to the protocols. The sequence of MO was as follows: Dre-miR-17a-MO, 5'-TACTACCTGCACTGTAAGCACTTTG-3'. For angiogenesis assays *in vivo*, Dre-miR-17a-MO (5 ng), Control-MO (5 ng) and NPC derived exosomes (5 ng) were injected into Tg (fli1a: EGFP) transgenic zebrafish 1-2-cell stage fertilized egg. 5 days after injection, fixed the embryos and analyzed green fluorescent signals using confocal microscopy (TCS-SP5 LSM, Leica, Germeny). Imaris software was used for analysis.

### Luciferase assay

We used online software TargetScan and Microcosm Targets to find potential downstream targets of has-miR-17-5p. Homo sapiens BMP and activin membrane bound inhibitor (BAMBI) oligonucleotides (66-73 bp), in which miR-17-5p binding sites were present (WT) or deleted (MUT), were inserted in the pGL3-Control vector to create recombinant plasmids according to the protocols. Luciferase activity was detected using HEK293 cells. After 48 h incubation, lysed the cells in cell culture luciferase lysis buffer and analyzed the luciferase activity using Dual-Luciferase Reporter Assay System (Promega, USA). The relative activity of luciferase was determined by the activity ratio of firefly luciferase and renilla luciferase.

### Statistical analysis

We used SPSS22.0 and GraphPad Prism 5 statistical software to analyze the data. One-way analysis of variance (ANOVA) was used to compare the expression of miR-17-5p in different tissues and cell lines, while χ^2^ test was used for multiple comparisons. The χ^2^ test and Student's t test were used to analyze the difference between groups. *p* < 0.05 indicated the difference was statistically significant.

## Results

### Upregulation of miR-17-5p promoted angiogenesis

**Figure 1:**
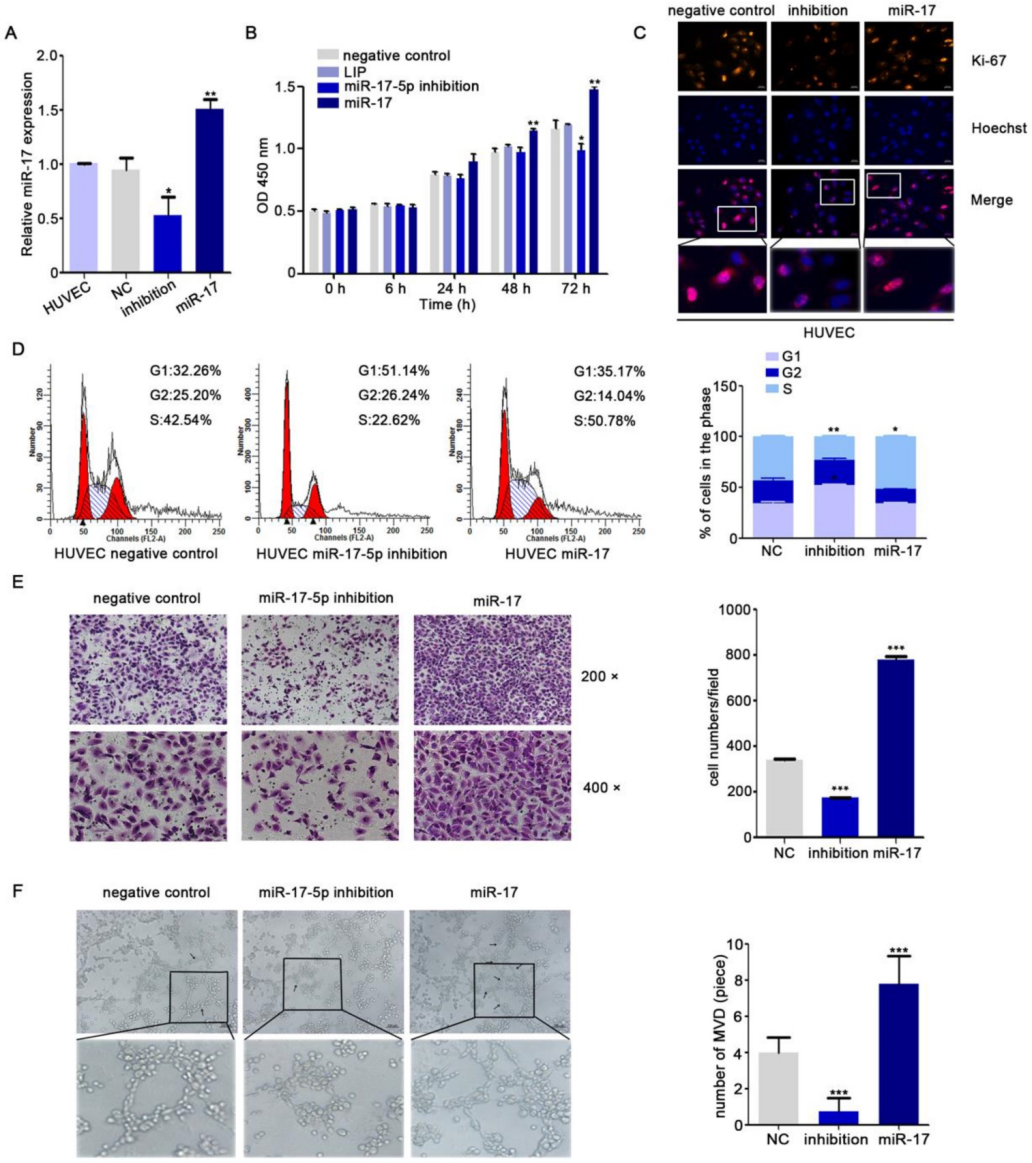
miR-17-5p regulates aniogenesis *in vitro*. **A:** Real-time PCR detected miR-17-5p expression after HUVECs transfected with plasmids. **B:** Cell viability of HUVECs was examined using CCK-8 assay after transfected with miR-17-5p inhibition plasmid, miR-17 plasmid and negative control.** C:** Ki-67 expression in HUVECs was detected by Immunofluorescence analysis. The bottom line was the amplification of the important parts of Merge. **D:** Cell cycle analysis showed the viability of HUVECs after altered miR-17-5p expression. **E:** Migration ability of HUVECs was analyzed using transwell assays. **F:** Representative images of capillary-like structure formed by HUVECs on Matrigel. The bottom line was the amplification of the capillary-like structure.

Yin R's research showed that miR-17-5p was closely associated with angiogenesis 35. To further explore the role of miR-17-5p in NPC angiogenesis, human umbilical vein endothelial cells (HUVECs) were transduced with different miR-17-5p plasmids (Fig. 1A). From the results of CCK8 assay and Immunofluorescence assay, we found that the proliferation ability of HUVECs was enhanced under the condition of excessive expression of miR-17-5p (Fig. 1B-C). Cell cycle analysis indicated that the percentage of HUVECs in G1 phase was increased after transfecting with miR-17-5p inhibition, while the S phase was increased significantly when miR-17-5p was upregulated (Fig. 1D). These data suggested that miR-17-5p could regulate the proliferation of HUVECs by influencing G1-S transition.

**Figure 2:**
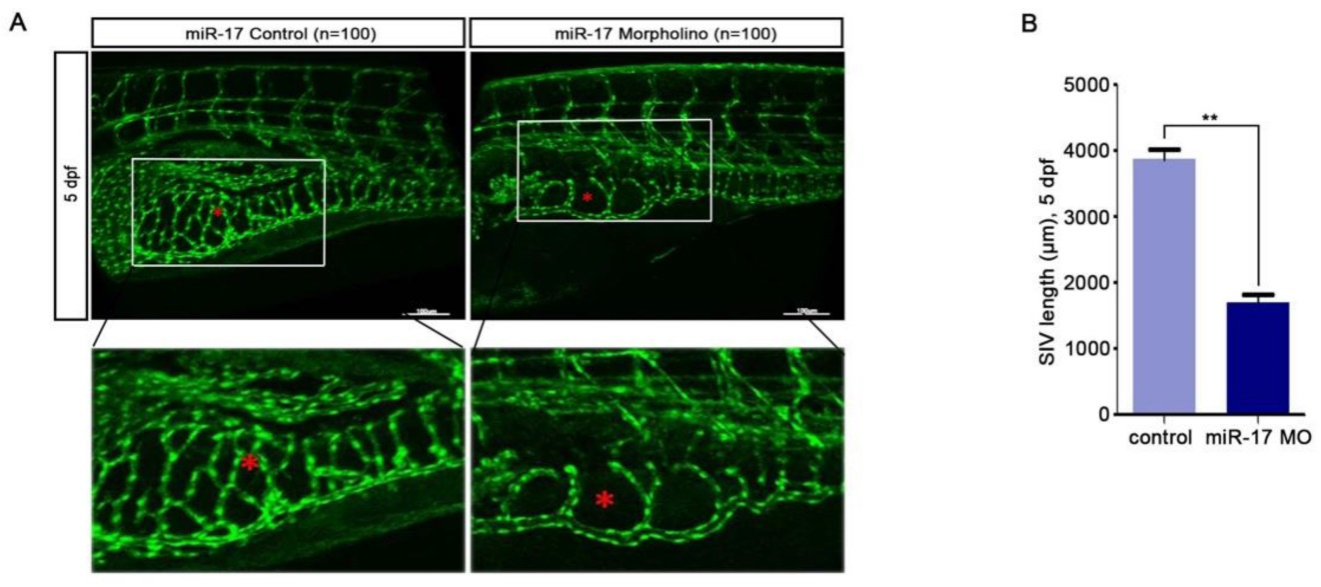
miR-17-5p regulates aniogenesis *in vivo*. **(A-B)**: Morphology of SIVs in 5 dpf Tg(fli1a:EGFP) embryos injected with miR-17 Morpholino and negative control. The bottom line was the amplification of SIVs. The data shown were representative of at least three independent experiments. * *p* < 0.05. ** *p*< 0.01.

For investigating the latent effects of miR-17-5p on angiogenesis, we studied whether miR-17-5p affected the migration ability of HUVECs. To our surprise, transwell assays showed significant inhibition of HUVECs migration after knockdown of miR-17-5p (Fig. 1E) and the results of tube formation indicated the acceleration effects of miR-17-5p on HUVECs (Fig. 1F). Given this significant change, we subsequently studied the role of miR-17-5p *in vivo*. After injected miR-17-5p morpholino and negative control into Tg (fli1a: EGFP) transgenic zebrafish embryos, embryos with low level of miR-17-5p caused abnormally length of blood vessels among subintestinal vessels (SIVs) during 5 days post fertilization (dpf) (Fig. 2). These results indicated that knockdown of miR-17-5p inhibited angiogenesis.

### Overexpression of miR-17-5p in NPC modulated cell proliferation and migration

**Figure 3:**
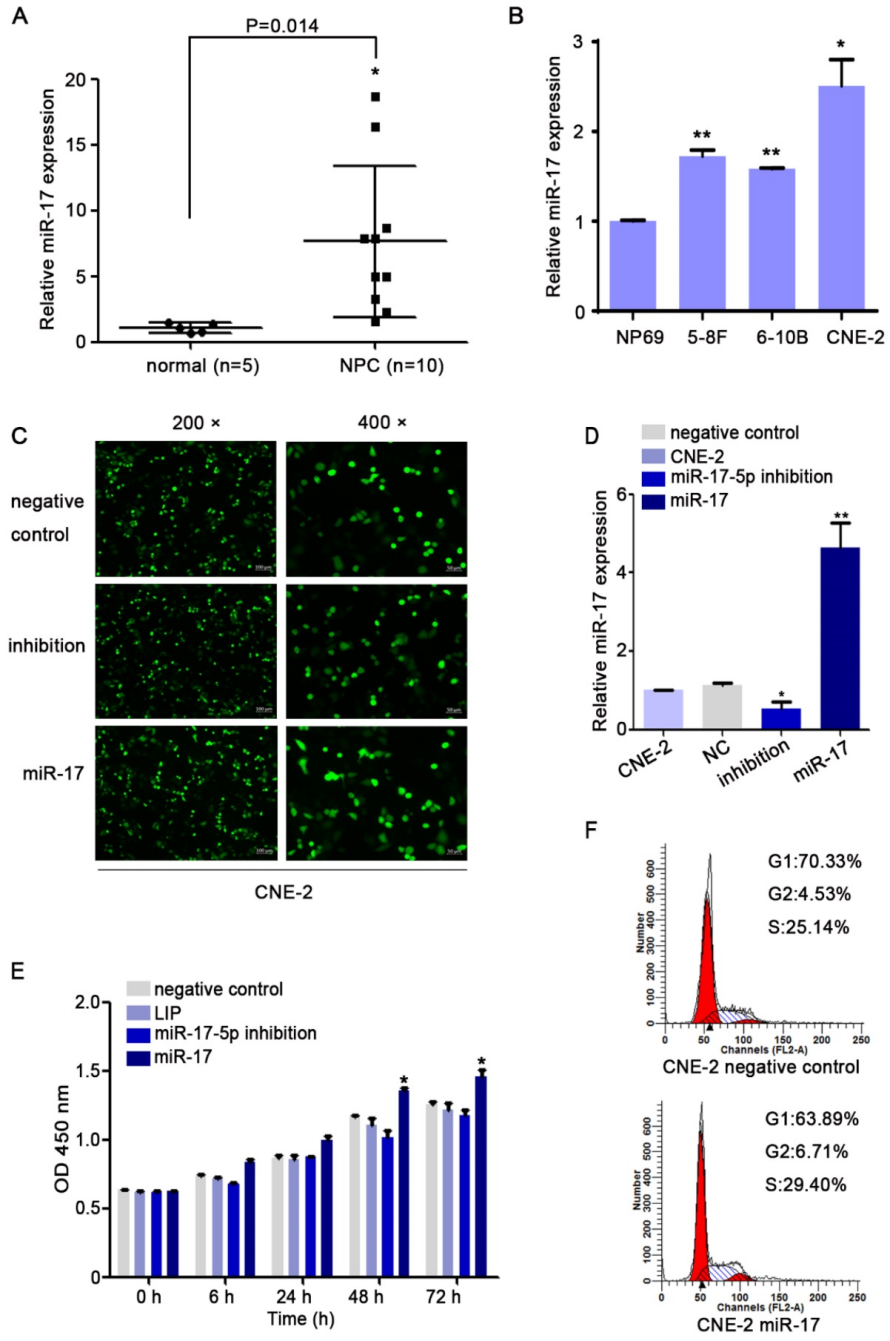
MiR-17-5p knockdown suppressed the proliferation of CNE2.** A:** MiR-17-5p expression in 10 NPC tissues and 5 normal nasopharyngeal tissues by Real-time PCR analysis.** B:** Relative miR-17-5p expression in NPC cell lines and normal nasopharyngeal epithelial cell line. U6 was an endogenous control.** (C-D):** Immunofluorescence analysis and Real-time PCR analysis of miR-17-5p expression after CNE-2 cells transfected with plasmids. **E:** Proliferation of CNE-2 cells was examined using CCK-8 assay after transfected with miR-17-5p inhibition plasmid, miR-17 plasmid and negative control.** F:** Cell cycle analysis by flow cytometry in CNE-2 cells containing different levels of miR-17-5p. The data shown were representative of at least three independent experiments. * *p* < 0.05. ** *p*< 0.01.

We used qRT-PCR to investigate the expression level of miR-17-5p. Our results indicated that compared with that in non-cancerous nasopharyngeal tissues, the miR-17-5p level in NPC was increased (Fig. 3A). Given this significant change, we further explored the expression of miR-17-5p in cell lines. The results showed higher expression of miR-17-5p in NPC cell lines than in NP69 (Fig. 3B). These data suggested that miR-17-5p is highly expressed in NPC.

As the highest miR-17-5p level in CNE-2 cells, we chose it for our subsequent experiments. To investigate miR-17-5p biological functions in NPC, CNE-2 cells were transfected with miR-17-5p inhibition plasmid, miR-17-5p plasmid and a negative control plasmid. As expected, miR-17-5p expression in cells was altered after being transfected by two different plasmids (Fig. 3C-D). CCK8 assay and cell cycle analysis demonstrated that after upregulated miR-17-5p expression, the proliferation rate and cell viability of CNE-2 cells were increased (Fig.3E-F).

**Figure 4:**
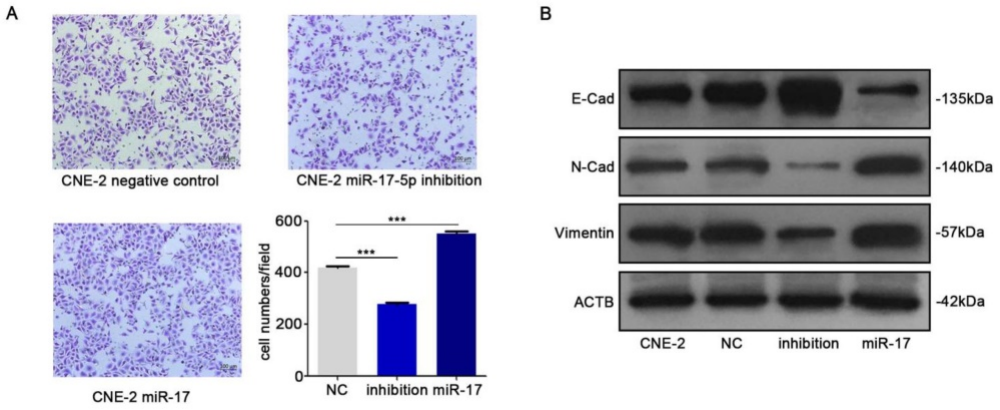
MiR-17-5p knockdown suppressed the migration of CNE-2.** A:** Using transwell assay to detect the penetration of CNE-2 cells transfected with plasmids through the membrane comparing with the control. **B:** Western blot showed the expression of EMT markers after CNE-2 cells transfected with miR-17-5p-specific plasmids and a negative control plasmid. β-actin was the loading control.

We subsequently studied the impact of miR-17-5p on the migration of CNE-2 cells. The results of transwell assay indicated that up-regulation of miR-17-5p could increase NPC cells migration (Fig. 4A). In order to further verify our results, we detected epithelial-mesenchymal transition (EMT) markers E-cadherin, N-cadherin and vimentin by western blot after upregulated the expression of miR-17-5p. Results indicated the facilitation of miR-17-5p in epithelial-mesenchymal transition of NPC cells (Fig. 4B).

The results above demonstrated that miR-17-5p facilitates the proliferation and migration of NPC cells.

### HUVECs ingested exosomal miR-17-5p derived from NPC cells

**Figure 5:**
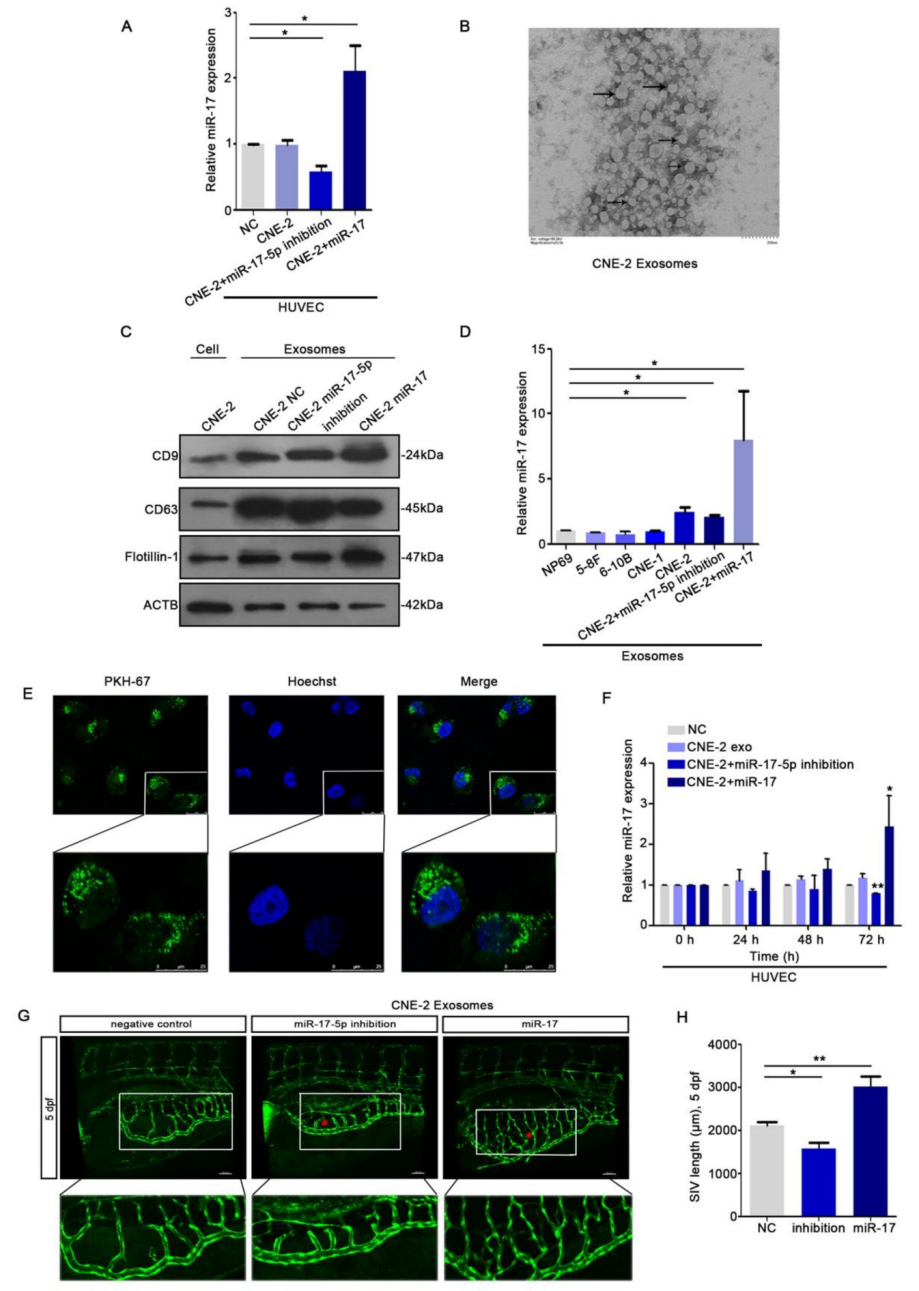
HUVECs ingested NPC derived exosomal miR-17-5p to promote angiogenesis.** A:** Real-time PCR analysed miR-17-5p expression in HUVECs which was co-cultured with CM from CNE-2 cells.** B:** Representative electron microscopy image of exosomes. **C:** Western blot showed the level of different kinds proteins in exosomes and CNE-2 cells. Flotillin-1 was the loading control. **D:** MiR-17-5p expression in exosomes was detected by Real-time PCR. **E:** Confocal microscopy images showed the ingestion of PKH67-labeled exosomes by HUVECs. Hoechst stained the nuclei (blue) and PKH67 labeled the exosomes (green). **F:** Real-time PCR showed miR-17-5p expression in HUVECs after absorbed exosomes from CNE-2 cells. **(G-H):** Morphology of SIVs in 5 dpf Tg(fli1a:EGFP) embryos injected with CNE-2 derived exosomes.

To explore whether there's any communication between HUVECs and NPC cells, we collected the conditioned medium (CM) from CNE-2 cells. We seeded HUVECs into the transwell chambers using CM from CNE-2 cell transduced with plasmids. The data of qRT-PCR indicated that HUVECs could ingest miR-17-5p from the CM (Fig. 5A). Since tumor-derived exosomes can release bioactive components acting on surface receptors of other cells, we next investigated the effects of NPC derived exosomes on NPC angiogenesis. We isolated exosomes from CNE-2 cell supernatant using ultracentrifugation. Transmission electron microscopy (TEM) showed structure with lipid bilayer membranes (Fig. 5B). Western blot showed that compared with whole CNE-2 cell lysates, purified exosomes were enriched with exosomal markers CD9 and CD63, while reduced of the cytoskeletal protein β-actin (Fig. 5C). Also, we found that the levels of miR-17-5p in exosomes secreted by NPC cell lines were all higher than normal (Fig. 5D).

To evaluate the effects of NPC exosomal miR-17-5p on angiogenesis, HUVECs were stimulated by exosomes with different levels of miR-17-5p. We found that HUVECs were able to ingest NPC derived exosomes (Fig. 5E), and the results of qRT-PCR analysis showed that the expression level of miR-17-5p in HUVECs stimulated by exosomes was changed over time (Fig. 5F). To verify the above findings *in vivo*, we subsequently injected exosomes with different levels of miR-17-5p into the Tg (fli1a: EGFP) transgenic zebrafish embryo for verification. Consistent with the results *in vitro*, exosomes enriched with miR-17-5p accelerated the length of blood vessels among embryo subintestinal vessels (SIVs) (Fig. 5G-H). All these data indicated that NPC derived exosomes can be ingested by HUVECs to promote angiogenesis.

### miR-17-5p targets BAMBI and regulates AKT/VEGF-A signaling

**Figure 6:**
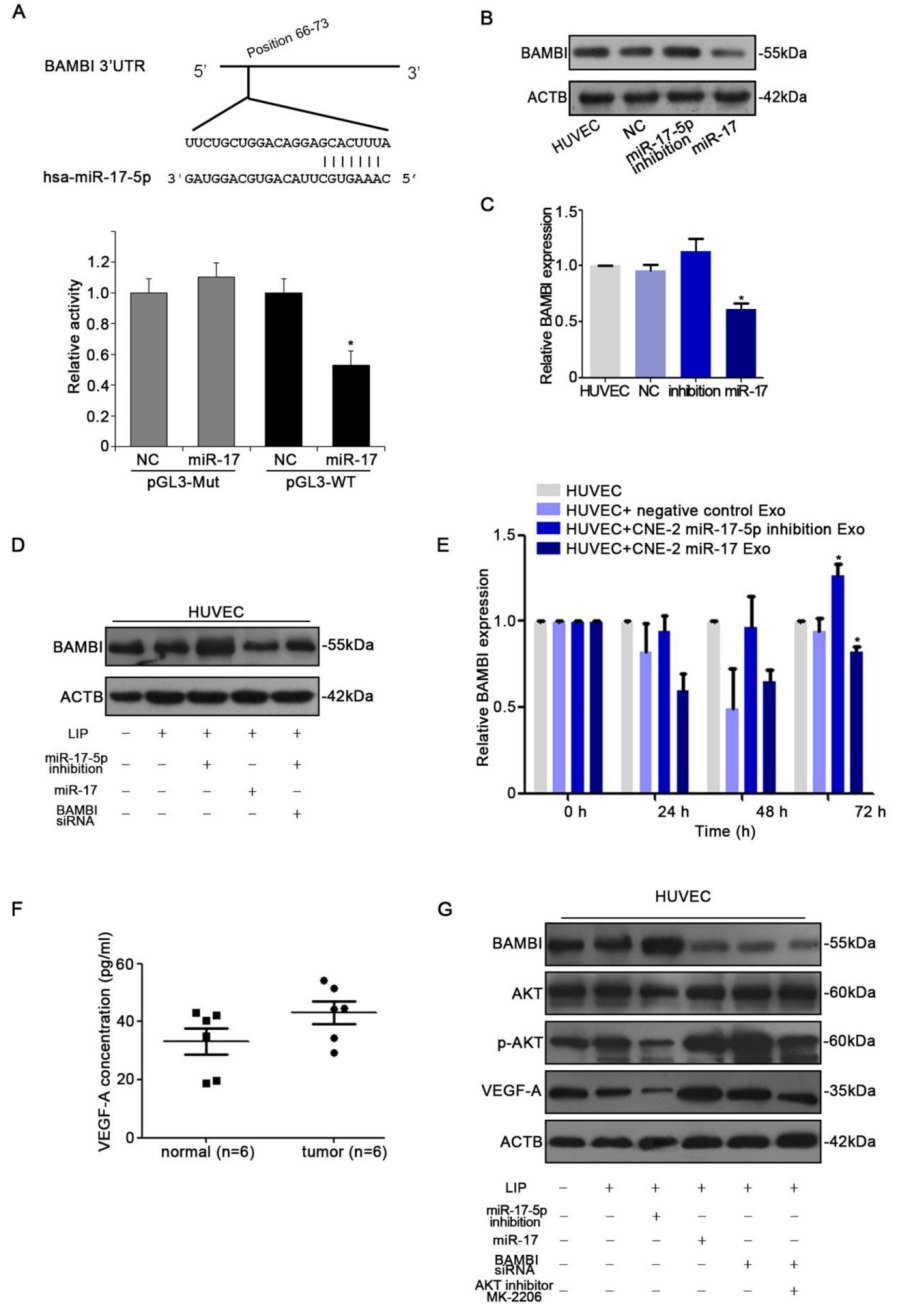
miR-17-5p targeted BAMBI expression and regulated AKT/VEGF-A signaling. **A:** Wild-type miR-17-5p target sequences of BAMBI mRNA 3'-UTR. Using luciferase reporter assays to quantitatively detect the relative luciferase activities of wild-type and mutant. **(B-D):** Quantifications of BAMBI mRNA and protein level in HUVECs using Real-time PCR and western blot. **E:** Real-time PCR detected BAMBI expression in HUVECs incubated with CNE-2 derived exosomes. **F:** Human VEGF-A Precoated ELISA Kit was used to measure serum VEGF-A levels in 6 NPC patients and 6 healthy controls. **G:** Western blot of BAMBI, p-AKT, AKT and VEGF-A expression in HUVECs. β-actin as the loading control. The data shown were representative of at least three independent experiments. * *p* < 0.05. ** *p*< 0.01.

With the observation of miR-17-5p inducing *in vitro* and *in vivo* angiogenesis, we next determined the target of miR-17-5p. Firstly, to identify putative miR-17-5p targets, TargetScan and Microcosm Targets were used. Among the hundreds of potential target genes, BAMBI was selected for the presence of potentially high binding sites, mediating tumorigenesis and angiogensis, and inhibiting TGF-β signaling which was reported to be regulated by miR-17-5p 36-38. Luciferase assays revealed that miR-17-5p repressed the activity of pGL3-REPORT-BAMBI-WT but not pGL3-REPORT-BAMBI-MUT (Fig. 6A). From the results of western blot and qRT-PCR, we found that alter the expression of miR-17-5p in HUVECs could thereby regulate BAMBI expression (Fig. 6B-D). We subsequently investigated whether the level of BAMBI in HUVECs would be changed after ingesting NPC derived exosomes enriched with miR-17-5p. qRT-PCR data showed that after HUVECs ingesting exosomes enriched with miR-17-5p, BAMBI expression was significantly downregulated, while the level of BAMBI showed an increasing trend after intaking of exosomes derived from CNE-2 cells transfected with miR-17-5p inhibition plasmid (Fig. 6E). These data indicated that BAMBI is a direct target gene of miR-17-5p.

To further investigate the molecular mechanism underlying NPC angiogenesis, we firstly used Human VEGF-A Precoated ELISA Kit to measure serum VEGF-A levels in 6 NPC patients with high expression of miR-17-5p and 6 healthy controls. The results showed higher level of serum VEGF-A as compared to controls (Fig. 6F). We thereby used western blot to further validate the relationship between BAMBI, AKT and VEGF-A. Western blot indicated that BAMBI can downregulate the expression of p-AKT and VEGF-A. At the same time, we found that using BAMBI-specific siRNAs to knockdown BAMBI expression can reverse this phenomenon (Fig. 6G). What's more, after added AKT signaling inhibitor MK-2206, the expression of BAMBI was not affected, while VEGF-A expression tended to decrease (Fig. 6G). Taken together, these findings suggested that exosomal miR-17-5p promoted tumor angiogenesis by downregulating BAMBI via AKT/VEGF-A signaling.

## Discussion

Although encouraging progress has been achieved in the research of molecular mechanism in NPC, the prognosis of patients with advanced NPC still unsatisfactory. Consequently, it is necessary to explore the deep mechanisms related to the occurrence and progression of NPC. Our research showed that miR-17-5p, expressed and secreted by NPC cells, plays a role in promoting tumor progression and angiogenesis by inhibiting the downstream target BAMBI. Also, the crosstalk between NPC cells and HUVECs by exosomal miR-17-5p in tumor microenvironment (TME) confirms that miR-17-5p may become a specific biological indicator for NPC and provide a new treatment strategy for it.

Studies have demonstrated that several dysregulated microRNAs in NPC tissues are involved in regulating the proliferation, migration and invasion of NPC cells. Studies have reported that miR-17 is involved in a variety of cancers, including gastric tumor, liver tumor, breast and lung tumor, etc., and plays an important role in regulating the biological characteristics of cancer cells 40. In Chen, C. previous studies, they considered miR-17-5p as a contributing factor to NPC development 41. We confirmed the result that miR-17-5p was upregulated in NPC cell lines. Functional investigations showed that knockdown of miR-17-5p suppressed NPC cell proliferation and migration. Also, suppression of miR-17-5p could reduce NPC angiogenesis.

Emerging evidence indicates that tumor microenvironment (TME) is not only the product of tumor development, but also one of the contributors that promote tumor development 42. As exosomes are important mediators of cancer-TME communication 43, we thereby explore their role in oncogenesis so that we can restrict their cancer-promoting features. MicroRNAs, the main RNA component of exosomes, play an important role in almost all aspects of oncology, such as tumorigenesis, proliferation, metastasis, chemoresistance and so on 20, 44. Secretory miRs are controlled by tumor cells and participate in the remodeling of TME 32,33. For example, exosomes enriched in miR-351b and miR-210 released by cancer cells increase tumor angiogenesis, thus promoting tumor metastasis 45. In our research, we detected that exosomal miR-17-5p increases HUVECs activity, suggesting that NPC cells can transmit genetic materials between cancer cells and HUVECs. Importantly, the overexpression of miR-17-5p in the serum of patients with NPC and the positive correlation between the serum containing miR-17-5p and tumor angiogenic activity suggested that exosomal miR-17-5p may be one of the main factors to promote tumor angiogenesis under pathological conditions.

BAMBI (BMP and Activin receptor Membrane Bound Inhibitor) is a transmembrane protein that is highly conserved in vertebrates from humans to zebrafish. As a competitive pseudoreceptor for members of TGF-β typeⅠ receptor family, BAMBI plays an important role in tumorigenesis and angiogenesis 37, 38. In this study, we used online databases to make predictions and further verified BAMBI as a potential target for miR-17-5p. After transfection of plasmid to suppress miR-17-5p expression, the level of BAMBI in HUVECs was increased, while the activity of angiogenesis was inhibited. We also confirmed that NPC derived miR-17-5p could reduce BAMBI expression via the transfer of exosomes, and increased AKT/VEGF-A expression in HUVECs. NPC cell derived exosomal miR-17-5p directly targeted the 3′UTR of BAMBI in HUVECs. All these data demonstrated that miR-17-5p played a role in promoting tumor development and angiogenesis via exosomal cell-cell interaction.

Taken together, our data indicated that miR-17-5p was highly expressed in NPC, which might play a role in promoting cancer progression in NPC. Overexpression of miR-17-5p could mediate angiogenesis. We also revealed the crosstalk between NPC cells and HUVECs, that was, exosomal miR-17-5p derived from NPC cells promoted angiogenesis. Therefore, all these results represented that miR-17-5p might serve as a new therapeutic target for the treatment of NPC.
